# Data for an Advanced Microstructural and Electrochemical Datasheet on 18650 Li-ion Batteries with Nickel-Rich NMC811 Cathodes and Graphite-Silicon Anodes

**DOI:** 10.1016/j.dib.2020.106033

**Published:** 2020-07-19

**Authors:** T.M.M. Heenan, A. Jnawali, M. Kok, T. G Tranter, C. Tan, A. Dimitrijevic, R. Jervis, D.J.L. Brett, P.R. Shearing

**Affiliations:** a Electrochemical Innovation Lab, Department of Chemical Engineering, UCL, London WC1E 7JE, UK; b The Faraday Institution, Quad One, Harwell Science and Innovation Campus, Didcot, OX11 0RA, UK

**Keywords:** Lithium-ion battery, Modelling, Electrode, Microstructure, Electrochemistry, X-ray computed tomography, Energy materials, Electric vehicle

## Abstract

The data presented here were collected from a commercial LG Chem cylindrical INR18650 MJ1 lithium-ion (Li-ion) battery (approximate nominal specifications: 3.5 Ah, 3.6 V, 12.2 Wh). Electrochemical and microstructural information is presented, the latter collected across several length scales using X-ray computed tomography (CT): from cell to particle. One cell-level tomogram, four assembly-level and two electrode/particle-level 3D datasets are available; all data was collected in the pristine state. The electrochemical data consists of the full current and voltage charge-discharge curves for 400 operational cycles. All data has been made freely available via a repository [10.5522/04/c.4994651] in order to aid in the development of improved computational models for commercially-relevant Li-ion battery materials.

Specifications table

**Table:** 

**Subject**	Electrochemistry
**Specific subject area**	This data covers the material properties and electrochemical performance of commercial Li-ion cells.
**Type of data**	Nine 2D radiograph sets (available upon request) as x-y-θ matrixes. Seven 3D volumes (.tif within repository) as x-y-z matrixes. One spreadsheet table (.csv within repository). Two 2D images (as Figs. 3 and 4 within the text).
**How data were acquired**	Samples: LG Chem INR18650 MJ1 cells (NKON, Netherlands) Electrochemical cycling: Maccor 4200 (Maccor Inc. U.S.A.) X-ray (full cell): Nikon XT H225 (Nikon Metrology, Inc. U.S.A.) X-ray (electrode assembly): Zeiss Xradia 520 Versa (Carl Zeiss., CA, USA) X-ray (electrode particles): Zeiss Xradia 810 Ultra (Carl Zeiss., CA, USA) Reconstructions: (full cell) ‘CT Pro 3D’,Nikon Metrology, Inc. U.S.A.; and (all other) ‘Reconstructor Scout-and-Scan’ (Carl Zeiss., CA, USA) Scanning electron microscope (SEM): EVO MA 10 SEM (Carl Zeiss, USA)
**Data format**	All raw
**Parameters for data collection**	Electrochemical: manufacturer's protocol (Table 1 & 2). Full-cell CT: 2278 projections using a polychromatic X-ray beam 58 keV (W-Kα), each with an exposure time of 1 s; with an isotropic 36.0 µm reconstructed voxel length. Electrode assembly CT (all four): 4500 projections using a polychromatic X-ray beam 58 keV (W-Kα), each with an exposure time of 10 s; with an isotropic 10.4 µm reconstructed voxel length. Printed Electrode CT (anode): 2400 radiograph projections using a quasi-monochromatic X-ray beam 5.4 keV (Cr-Kα), each with an exposure time of 30 s; with an isotropic 63.1 nm reconstructed voxel length. Printed Electrode CT (cathode): 1200 radiograph projections using a quasi-monochromatic X-ray beam 5.4 keV (Cr-Kα), each with an exposure time of 60 s; with an isotropic 63.1 nm reconstructed voxel length. SEM imaging: all parameters stated on Figs. 3 and 4.
**Description of data collection**	Electrochemical data was collected by either controlling the cell current or voltage. X-ray data were collected by sequentially exposing the sample to the X-ray beam in order to collect 2D radiographs that are later reconstructed into 3D tomograms. SEM images were collected through a rastered electron-beam across the sample.
**Data source location**	UCL Data Repository Electrochemical Innovation Lab, University College London, Gower Street, London, WC1E 6BT
**Data accessibility**	Repository name: UCL Data Repository UCL RDR Data identification number: [10.5522/04/c.4994651] Direct URL to data: [http://doi.org/10.5522/04/c.4994651]

## Value of the data

• This data provides a multi-length scale microstructural analysis of a Li-ion cell.
• In combination with the complementary electrochemical data, this may be used for advanced computational modelling.
• Supply of data on both charge and discharge, in combination with both anode and cathode microstructures, allow for the lithiation, and de-lithiation processes to be explored.
• Complex computational models may extend our knowledge of cell degradation and lifetime.

## 1 Data description

**Fig. 1:**
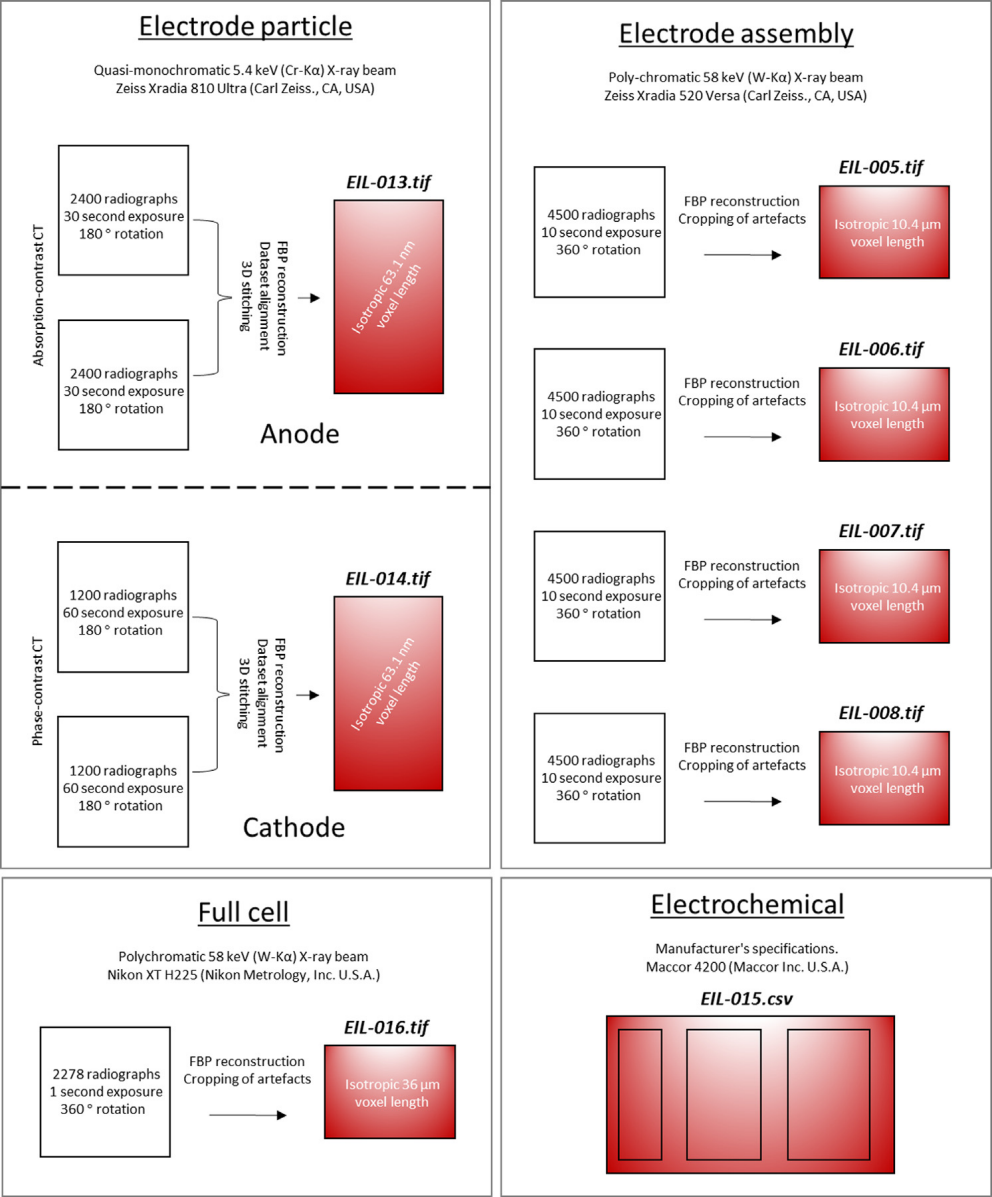
Information on the data made available: particle- to cell-level 3D data and electrochemical cycling data (Red datasets are available via the repository).

**Fig. 2:**
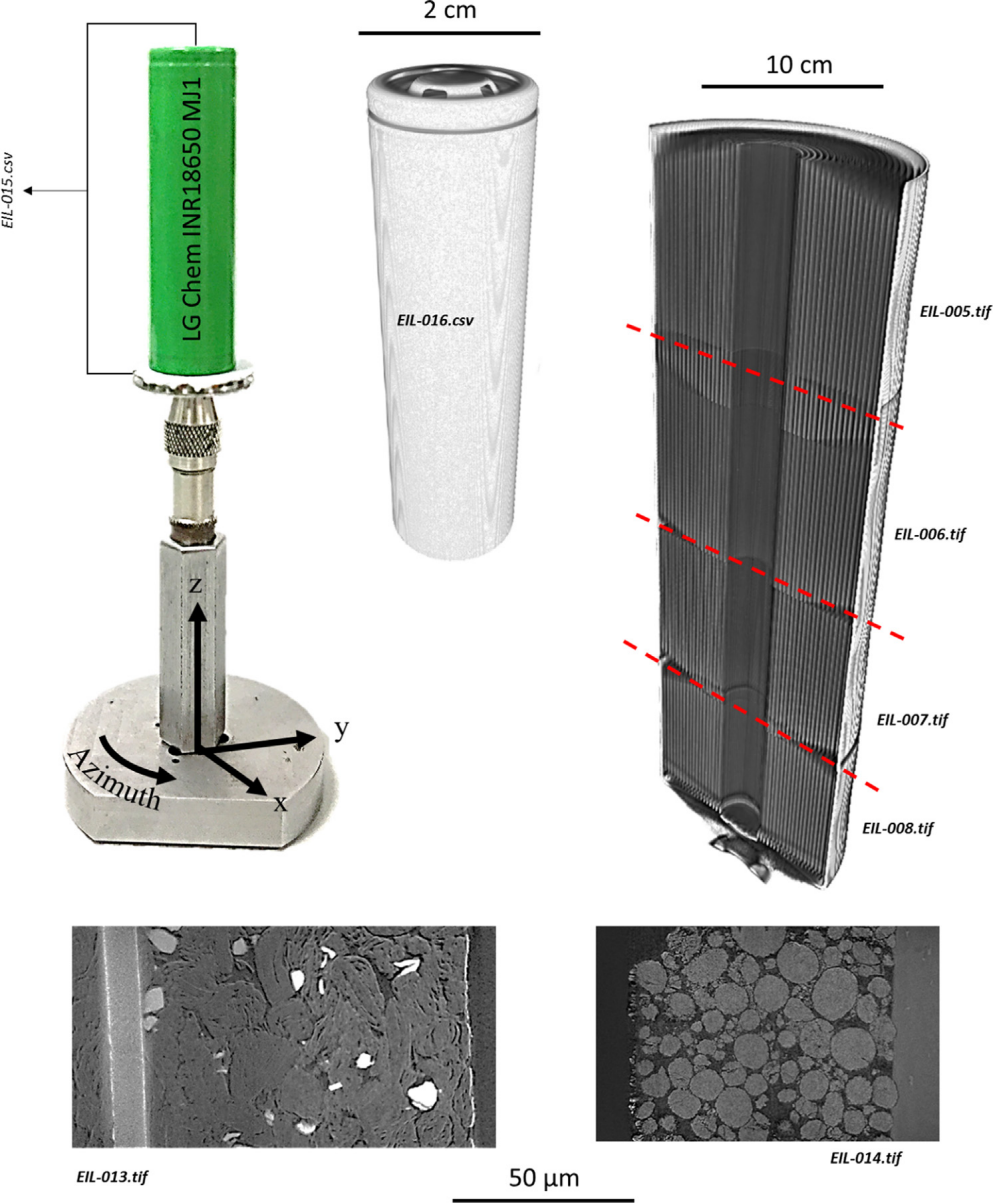
Visualisation of the data made available through this work.

The data described within this article and made available via the repository are outlined in Fig. 1, with the reconstructed data displayed within Fig. 2. The data collected from the samples can be summarised as follows.

One full-cell data set in the form of a 3D .*tif* file: EIL-016.

Four electrode assembly data sets in the form of 3D .*tif* files: EIL-005; EIL-006; EIL-007; EIL-008.

Two electrode particle data sets in the form of 3D .*tif* files: EIL-013; EIL-014.

One spreadsheet table containing electrochemical cycling data in .*csv* format: EIL-015.

**Fig. 3:**
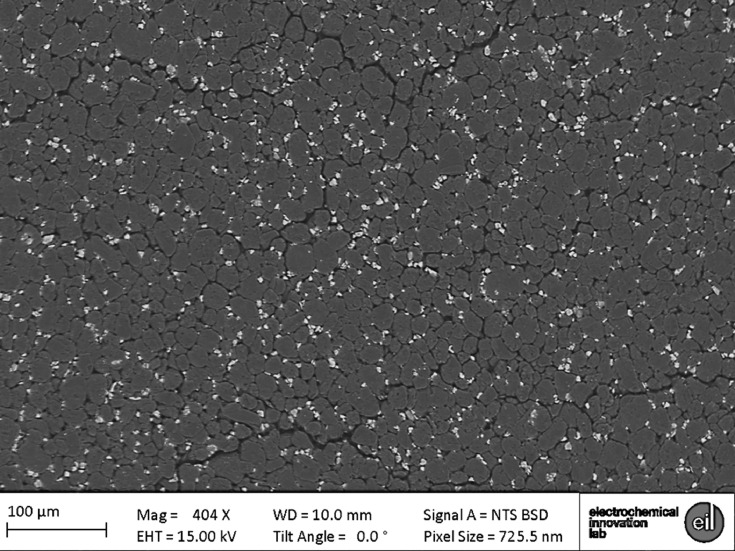
MJ1 anode SEM image collected using an EVO MA 10 SEM (Carl Zeiss, USA).

**Fig. 4:**
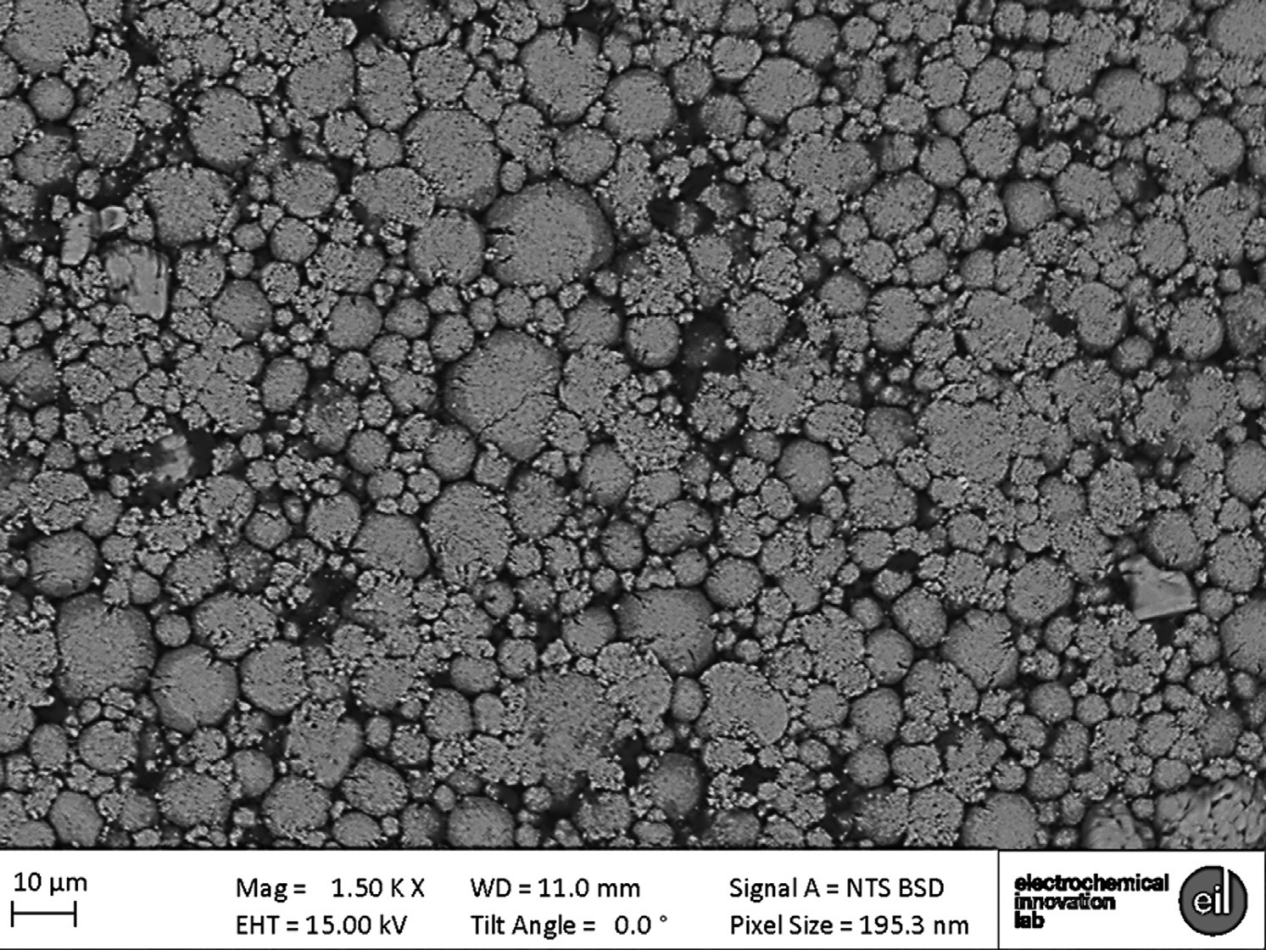
MJ1 cathode SEM image collected using an EVO MA 10 SEM (Carl Zeiss, USA).

To complement the microstructural data, SEM images are also supplied within this article in Figs. 3 and 4. It should be noted that all 3D microstructural data presented within this article are in the pristine state, i.e. was collected prior to electrochemical cycling, as purchased. Only reconstructed data is published within the repository (i.e. EIL-005, 006, 007, 008, 013, 014 and 016); however, all pre-reconstruction data can be made available upon request from the corresponding authors.

## 2 Experimental design, materials, and methods

All data in this work were obtained from commercial LG Chem INR18650 MJ1 cells (NKON, Netherlands) and the manufacturer's specifications can be found within Tables 1 and 2. Although not noted on the manufacturer's specifications, it has been previously stated that the cathode and anode consist of NMC811 (LiNi_0.8_Mn_0.1_Co_0.1_O_2_) and SiO_x-_C, respectively [1], [2], [3].

**Table 1:** Manufacturer's (LG Chem) nominal specifications for INR18650 MJ1 cells.

Model	INR18650 MJ1
Capacity	Nominal 3.5 Ah Min. 3.4 Ah
Voltage	Avg. nominal 3.635 V Top of charge 4.2 V Bottom of discharge 2.5 V
Charging currents	Standard current 0.5 C (ca. 1.7 A) Max. current 1.0 C (ca. 3.4 A) Cut-off current 50 mA
Discharging currents	Standard current 0.2 C (ca. 0.68 A) Max. current 3.0 C (ca. 10 A)
Operating temperatures	Charge range 0 ∼ 45 °C Discharge range −20 ∼ 60 °C Standard 23 ± 2 °C
Relaxation periods	10 mins after charge 20 mins after discharge
Storage temperatures	1 month −20 ∼ 60 °C 3 month −20 ∼ 45 °C 1 year −20 ∼ 20 °C
Weight	Max. 49.0 g
Diameter	Avg. 18.4 + 0.1 – 0.3 mm Max. 18.5 mm
Height	Avg. 65 +−0.2 mm Max. 65.2 mm

**Table 2:** High drain charge/discharge conditions.

Model	INR18650 MJ1
Charge CC	1.5 A to 4.2 V
Charge CV	4.2 V until end current of 100 mA
Top of charge rest	10 min
Discharge CC	4.0 A to 2.5 V
Discharge CV	None – N/A
Bottom of charge rest	20 min

The full-cell X-ray radiographic data was collected using a Nikon XT H225 instrument (Nikon Metrology, Inc. U.S.A.) with an accelerating voltage of 170 kV_p_, with a stationary tungsten anode that produces a polychromatic beam with a characteristic emission peak at 58 keV (W-Kα). One full-cell CT scan was collected via 2278 projections and an exposure time of 1 s. The tomogram (EIL-016) was reconstructed using commercial software employing cone-beam filtered back-projection algorithms (‘CT Pro 3D’, Nikon Metrology, Inc. U.S.A.). The magnification produced an isotropic voxel length of 36.0 µm.

The electrode assembly X-ray radiographic data were collected using a 520 Versa X-ray CT instrument (Zeiss Xradia 520 Versa, Carl Zeiss., CA, USA) with an accelerating tube voltage of 120 kV_p_, with a stationary tungsten anode on a copper substrate that produces a polychromatic beam with a characteristic emission peak at 58 keV (W-Kα). Four CT scans were conducted in total, each with 4500 X-ray radiograph projections collected per scan and a 10 s exposure per projection. After acquisition, the four sets of 2D macro-CT radiographs were reconstructed into four 3D tomograms (EIL-005, EIL-006, EIL-007 and EIL-008) using commercial software employing cone-beam filtered back-projection algorithms (‘Reconstructor Scout-and-Scan’, Carl Zeiss., CA, U.S.A.). The magnification produced an isotropic voxel length of 10.4 µm in all four datasets.

The nano-CT radiographs were collected using an 810 Ultra X-ray-CT instrument (Zeiss Xradia 810 Ultra, Carl Zeiss., CA, USA) with an accelerating tube voltage of 35 kV_p_ that employs a rotating chromium anode. This produces a quasi-monochromatic beam with a characteristic emission peak of 5.4 keV (Cr-Kα). A capillary condenser produces focused X-rays for a full-field illumination of the sample, projected onto the scintillator detector using a Fresnel zone-plate. Two samples were inspected: one MJ1 anode and one MJ1 cathode. The cathode was imaged using Zernike phase-contrast mode, where a phase-ring was inserted to emphasize edge features, whereas the anode was imaged without the use of the phase ring, i.e. absorption dominated, in order to maximize the contrast between the SiO_x_ and the carbon. Moreover, in order to capture the full electrode thicknesses, two CT scans were conducted on each sample. The two absorption-contrast nano-CT scans of the anode required 2400 X-ray radiograph projections per scan, with a 60 s exposure time per projection. The two phase-contrast nano-CT scans of the cathode required 1200 X-ray radiograph projections per scan, with a 30 s exposure time per projection. The four nano-CT datasets were then reconstructed using commercial software employing parallel-beam filtered back-projection algorithms (‘Reconstructor Scout-and-Scan’, Carl Zeiss., CA, U.S.A.), producing an isotropic voxel length of 63.1 nm. The reconstructed volumes were then stitched using Avizo Fire software (Avizo, Thermo Fisher Scientific, Waltham, Massachusetts, U.S.A.) producing one nano-CT tomogram for the anode (EIL-013) and one for the cathode (EIL-014). Both the anode and cathode tomograms are available via the repository. The visualization for Fig. 2 was also achieved using Avizo Fire software.

Electrochemical cycling was achieved using a Maccor 4200 cycler (Maccor Inc. U.S.A.). Charging was performed at a constant current of 1.5 A until 4.2 V, then the voltage was held until the current reached 100 mA. Discharging was performed at 4.0 A to 2.5 V. This protocol was followed for 400 cycles (as recommended by the manufacturer's high drain protocol Table 2). All cycling was performed within an environmental chamber set to 24 °C, although cell temperatures were recorded to increase above this, particularly during points of high current due to Joule heating. The data was exported into a .csv file (EIL −015, available via the repository).

Scanning electron microscope imaging was conducted using an EVO MA 10 SEM (Carl Zeiss, USA). Images of both the anode and cathode were collected with accelerating voltages of 15 kV and a working distance of 10 mm and 11 mm, respectively. These are presented in Figs. 3 and 4.

## Author contributions

TH and RJ collected of the nano-tomography data. TH and TT collected the macro-CT data. TH and MK processed the tomography data for dissemination. AD collected the SEM data. AJ collected the electrochemical data. TH, AJ and CT processed the electrochemical data for dissemination. TH, DJLB and PRS directed all research. All authors reviewed the article.

## Declaration of Competing Interests

The authors declare that they have no known competing financial interests or personal relationships which have, or could be perceived to have, influenced the work reported in this article.
